# Current challenges and future perspectives for patient safety in surgery

**DOI:** 10.1186/1754-9493-8-9

**Published:** 2014-02-21

**Authors:** Philip F Stahel, Cyril Mauffrey, Nathan Butler

**Affiliations:** 1 Department of Orthopaedics, Denver Health Medical Center, University of Colorado Denver, School of Medicine, 777 Bannock Street, Denver, CO 80204, USA

## 

As surgeons, we are arguably practitioners of one of the most entitled, rewarded and rewarding occupations in the world. We are privileged to meet and interact with previously unknown individuals on a most intimate and personal level, and to make a positive difference at some of the worst times in their lives. We eventually know these people in ways they cannot know themselves, and we are able help them in ways they cannot help themselves. We are empowered to the completely legal action of putting a knife to work in a human body. With proper indication and distinguished technical skills, our surgical blade can provide a cure for acute and chronic ailments in the most vulnerable population of human beings. In return, our patients reward us with their unlimited trust in our knowledge, skills, and ability to deliver them to restored health and an improved quality of life. Unfortunately, we fail to restore our patients’ health and quality of life more often than we appreciate. While all physicians take the Hippocratic Oath to abstain from doing harm (*”Primum non nocere”*), our patients are frequently caught in the ‘friendly fire’ of surgical care – health care providers causing unintentional harm when their only intent was to help [1,2].

Interestingly, adverse events resulting from surgical interventions are actually more frequently related to errors occurring before or after the procedure than by technical mistakes by a surgical blade ‘gone wrong’. These include *(i)* breakdown in communication within and amongst the surgical team, care providers, patients and their families; *(ii)* delay in diagnosis or failure to diagnose; and *(iii)* delay in treatment or failure to treat [3-5]. On a daily basis, surgeons must adjudicate challenges that reach far beyond pure technical aspects – the decision of initiating appropriate and timely surgical care, weighed against the risk of providing delayed or negligent care by rather choosing observation and/or non-operative treatment. This narrow margin represents the foundation of a surgeon’s eternal ‘moment of truth’ (*“to cut or not to cut”*) which could be a crucial turning point in the long-term future of our patients.

How can patients be sure that their surgeon is competent, knowledgeable, and well trained? How can patients be sure that the proposed treatment modality or surgical procedure represents the optimal treatment of choice? How can patients be sure that surgeons are singularly incentivized to provide only high quality and safe surgical care, independent of other metrics of success, including entrenched financial interests? How can patients be sure that the surgical team is dominated by an immutable *‘culture of patient safety‘* with full buy-in by all members of the team? How can patients be sure that they will not be exposed to the learning curve of a new procedure or a young surgeon in training?

Ironically, the high standard of regulatory compliance-mandated patient safety protocols in the United States emanates from decades of work by lawyers and patient advocacy groups, not from physician-driven initiative. It is time to end this historic negligence. It is time for surgeons to direct and own patient safety as a ‘surgical responsibility’.

More than 200 million surgeries are performed worldwide each year [6]. Any patient admitted to a hospital to undergo a surgical procedure should rightfully expect to be better off after the intervention than before. However, recent reports reveal that adverse event rates for surgical conditions remain unacceptably high, despite multiple nationwide and global patient safety initiatives over the past decade [7]. These include the ’100,000 Lives Campaign’ (2005/2006) and subsequent ‘5 Million Lives Campaign’ (2007/2008) by the Institute for Healthcare Improvement (IHI), the ‘Surgical Care Improvement Project’ (2006) and ‘Universal Protocol’ (2009) by the Joint Commission, and the WHO ‘Safe Surgery Saves Lives’ campaign accompanied by the global implementation of the WHO surgical safety checklist (2009) [8-13].

Many of the current limitations to the creation of a globally recognized and consistently practiced *‘culture of patient safety’* stem from the lack of surgeon-driven leadership. Transparent leadership and credible role modelling are the prerequisites to ensure unwavering ‘buy-in’ by all members of the health care team for adoption of safety practices in the daily routine, including strict adherence to patient safety checklists and safety core measures [14]. We are furthermore lacking a uniform system for reporting and analysis of surgical complications, which could be modelled on the *Problem Reporting and Corrective Action* (PRACA) quality assurance database by the National Aeronautics and Space Administration (NASA) [15]. Errors in the surgical care of our patients frequently lead to unintentional harm on first occurrence in absence of a ‘fail-safe’ backup option. We should learn from other high-risk domains, including nuclear technology, professional aviation, naval submarine technology, and aerospace engineering that have historically embraced a culture of safety as a basic tenet for the success in their respective missions. In engineering, ‘redundancy’ implies the ‘fail-safe’ duplicate or triplicate availability of critical components or system functions. For example, NASA endorses the fundamental principle of being ‘double-fail-safe’ in all aspects of their enterprise [15].

Patient safety in surgery should model on the 5 core principles from NASA’s proven safety culture paradigm:

1. *Reporting culture* – Reporting concerns without fear of reprisal.

2. *Learning culture* – Learning from successes and failures.

3. *Flexible culture* – Changing and adapting to meet new demands.

4. *Engaged culture* – Everyone is doing their part.

5. *Just culture* – Treating each other fairly.

The extrapolation of these proven safety pillars from aerospace engineering to patient safety in surgery is challenged by multiple barriers imposed by our current health care system. Based on the premise that *“Good judgment comes from experience which comes from poor judgment”* (Figure 1), NASA’s safety culture originated from lessons learned through system failure analysis after dramatic fatal accidents, including the Apollo 1 cabin fire in 1967, and the space shuttle disasters in 1986 and 2003. In surgery, we are still falling short of implementing a formal *‘culture of reporting and learning’*.

**Figure 1:**
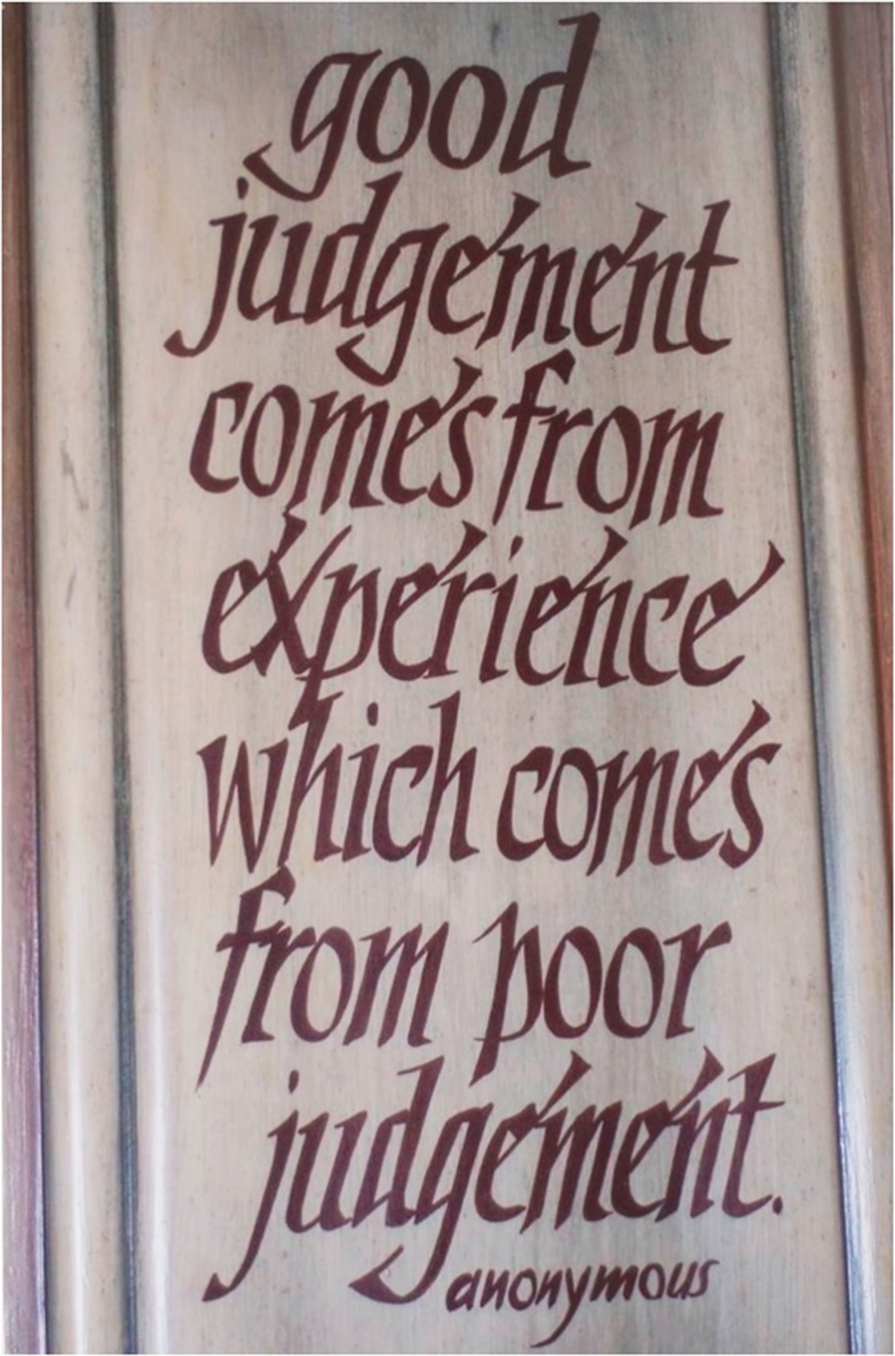
Paradigm of the learning curve in surgery and other high-risk domains.

In the absence of the long overdue legislative tort reform needed to avoid penalties for publicly reporting medical errors, surgeons remain understandably reluctant to disclose surgical complications in an open and transparent forum [16,17]. The deterrent of potential punitive measures could be mitigated by adopting a model from professional aviation safety, such as the amnesty program used by the U.S. Federal Aviation Administration (FAA). The FAA program is designed to incentivize pilots and air traffic controllers to report poor personal conduct, including sleeping on duty or falsifying records. The FAA claims that since the implementation of the amnesty program *“No other safety program has identified and fixed more local and systemic problems in any other high-risk domain”*[18].

In medicine, the absence of formal amnesty programs combined with the daunting threat of legal repercussions for admitting and reporting errors and complications, appears to breed a converse *‘culture of silence and intolerance’*. The current pressure of the medicolegal industry furthermore promotes a *‘culture of defensive medicine’* by setting a standard expectation for diagnostic precision that borders on fantasy. The unintentional fallout from practicing defensive medicine is a drastic exacerbation of health care costs, with little or no benefit to the patient, in conjunction with an increased risk for ‘collateral damage’ by the overuse of diagnostic testing [19,20]. For example, the exponentially increased use of medical imaging by computed tomography scans in recent years has been associated with an incremental long-term risk of radiation-induced cancer [21-23]. Further unresolved problems include the wide variation of surgical indications worldwide, the inequity of access to surgery for disparities, and a questionable long-term sustainability of surgical quality at the current rate of progress associated with increasing costs for modern and innovative procedures [6,14].

An additional serious challenge to patient safety in surgery consists of the questionable quality of training for the next generation of surgeons. The desperate need for more primary care doctors in the coming years and decades prompted selected medical schools in the United States to shorten their teaching curriculum to just 3 years by shaving off one full year of training [24]. This ’fast-track MD’ program is certainly appealing by saving tuition costs and addressing the predicted shortage of primary care physicians. However, cutting the training curriculum of new physicians appears rather counter-intuitive from a patient safety and quality perspective. Additionally, the surgical experience of residents in training has been drastically impaired by the implementation of resident work hour restrictions [25-28]. Ironically, work hour restrictions were implemented as a patient safety measure to mitigate the risk of surgical complications originating from overworked and fatigued residents. Contrary to the original intent, a decade of international experience with resident work hour restrictions revealed that patients are not safer, but rather more susceptible to harm originating from handovers of care, equivocal physician accountability, and breakdowns in communication within the team [28-34]. In addition, multiple studies on millions of hospital admissions in different countries reported a lack of an effect of resident work hour restrictions on patient morbidity and mortality, bringing into question the primary intent of the program in the first place.

Surgeons are under an increasing amount of pressure and expectation to perform at the highest level. They must deliver absolute diagnostic accuracy and infallible surgical quality under the conflicting paradigm of patient safety and maximal cost efficiency. In addition, surgeons are expected to have the highest standards of ethical values and professionalism, to act as respected role models, dedicated teachers, academic researchers, successful administrators and entrepreneurs. While no medical student would ever learn about managing a business during medical school, surgeons are increasingly requested to provide cost-efficient care under an increasingly competitive ‘health care market’. These expectations come close to the task of squaring the circle even for experienced surgeons, but are virtually unattainable for physicians in training who are denied adequate access to surgical ‘hands-on’ training in the current age of work hour restrictions and ‘fast-track’ teaching curricula. We are worried that the next generation of surgeons may not have an adequate opportunity of learning ’how to cut’ and may have to postpone the learning curve from training (Figure 1) to an unsupervised surgical practice in later years. This is certainly not in the patients’ best interest.

An intuitive solution, in light of the demonstrated absence of a positive effect of resident work hour restrictions on patient safety and outcomes, is for accreditation councils of residency programs to reconsider the value and far-reaching consequences of work hour restrictions, and to potentially drop this inefficient program. In addition, it is our obligation as senior surgeons to act as role models to our trainees with regard to professionalism and individual physician accountability, and to prove these values in daily interactions with our team [35]. As we observed the historic paradigm shift from a *‘culture of blame and shame’* to a *‘culture of systems safety’*, we have now reached a tipping point in which the expectation of systems are exhausted, and a physician-driven approach is needed to build and sustain a *‘culture of individual accountability’*. A classic example is hand hygiene as a simple core measure with immense impact on patient safety with regard to decreasing the incidence of hospital-acquired infections. International estimates show that overall compliance with hand hygiene among health care personnel is as low as 5% to 30% [36-38]. A ‘perfect’ system can provide staff training programs and logistic support, including door signs, checklists, and hand sanitizer dispensers in- and outside of patient rooms. However, in absence of individual accountability and physician-driven leadership, the expected goal of 100% hand hygiene compliance remains utopic. How is it possible that low-wage workers in the meat packing industry are able to sustain 100% compliance with hand hygiene protocols, but physicians can’t? Intriguing insights from our own institution reveal that hand hygiene compliance rates drop from more than 90% when officially observed and monitored, to less than 40% when we feel unobserved. This phenomenon likely relates to the ‘Hawthorne effect’ by which a subject’s behavior changes as a result of being observed, and reflects poorly on the physicians’ accountability for *‘doing the right thing’* for our patients at all times.

On a positive side, the historic dogma that physicians are infallible has worn off and has been replaced by a modern concept of patient-centered care, with patient safety as its core tenet. The concept of involving patients and families in a ‘shared decision-making’ approach for surgical care has globally evolved in recent years as a cornerstone of patient-centered care (*“Nothing about me without me!”*) [39]. Despite all limitations and barriers outlined in this editorial which continue to impede the implementation of a sustainable and global *‘culture of patient safety’*, we are extremely positive that the future for our patients is bright! We see the bright future every day in the eyes of our trainees, medical students and residents, in their unlimited enthusiasm and proactive engagement in all aspects related to patient safety, quality assurance and quality improvement. The only benchmark for our success as mentors is to produce trainees who will be better surgeons and stronger patient safety advocates than we could have ever been in our own life time [40,41].

The legendary Flight Director of the lunar Apollo missions, Gene Kranz, stated in the wake of the Apollo 1 disaster in 1967 [15]:

“From this day forward, Flight Control will be known by two words: ‘Tough and competent’. Tough means that we are forever accountable for what we do or what we fail to do. We will never again compromise our responsibilities. Competent means we will never take anything for granted. We will never be found short in our knowledge and in our skills.”

It is time for surgeons to become *‘tough and competent’* in patient safety!

## Competing interests

All authors are members of the *Patient Safety in Surgery* editorial board. The authors declare no financial conflict of interest related to this manuscript.

## Authors’ contribution

PFS designed and drafted the first version of this editorial. All authors contributed equally to revisions of the manuscript. All authors read and approved the final version of the editorial prior to submission.
